# *In vitro* inhibitory effects of bergenin on human liver cytochrome P450 enzymes

**DOI:** 10.1080/13880209.2018.1525413

**Published:** 2018-12-04

**Authors:** Gang Dong, Yun Zhou, Xiaoli Song

**Affiliations:** Department of Pharmacy, Yidu Central Hospital of Weifang, Shandong, China

**Keywords:** CYP3A4, CYP2E1, CYP2C9, herb–drug interaction

## Abstract

**Context:** Bergenin, isolated from the herb of *Bergenia purpurascens* (Hook. f. et Thoms.) Engl., has anti-inflammatory, antitussive, and wound healing activities. However, whether bergenin affects the activity of human liver cytochrome P450 (CYP) enzymes remains unclear.

**Materials and methods:** In this study, the inhibitory effects of bergenin (100 μM) on the eight human liver CYP isoforms (i.e., 1A2, 3A4, 2A6, 2E1, 2D6, 2C9, 2C19 and 2C8) were investigated, enzyme kinetics and time-dependent inhibition studies were also performed *in vitro* using human liver microsomes (HLMs).

**Results*:*** The results showed that bergenin inhibited the activity of CYP3A4, 2E1 and 2C9, with IC_50_ values of 14.39, 22.83 and 15.11 μM, respectively, but other CYP isoforms were not affected. Enzyme kinetic studies showed that bergenin was not only a non-competitive inhibitor of CYP3A4, but also a competitive inhibitor of CYP2E1 and 2C9, with *K_i_* values of 7.71, 11.39 and 8.89 μM, respectively. In addition, bergenin is a time-dependent inhibitor for CYP3A4 with *K*_inact_/*K_I_* value of 0.025/3.50 μM^−1 ^min^−1^.

**Discussion and conclusions:** The *in vitro* studies of bergenin with CYP isoforms indicate that bergenin has the potential to cause pharmacokinetic drug interactions with other co-administered drugs metabolized by CYP3A4, 2E1 and 2C9. Further clinical studies are needed to evaluate the significance of this interaction.

## Introduction

*Bergenia purpurascens* (Hook. f. et Thoms.) Engl., a traditional Chinese medicine, possesses anti-inflammation and antidiarrhoeal abilities and is used clinically for the treatment of diarrhoea, dysentery and other gut-associated diseases (Shi et al. 2014; Pandey et al. 2017; Liu et al. 2018). Bergenin is the major bioactive ingredient in the herb–drug (Li et al. 2013; Pandey et al. 2017). Therapeutically, it is used as an antiarrhythmic, antifungal, anticancer, antidiabetic and antioxidant agent (Bessong et al. 2005; Ahmed and Urooj 2012; Bajracharya 2015; Aggarwal et al. 2016).

Cytochrome P450 (CYP450) enzymes are important phase I enzymes in the biotransformation of xenobiotics, and CYP450 enzymes can be inhibited or induced by a variety of drugs and chemicals that can give rise to toxicity or treatment failure (Wrighton and Stevens 1992; Li 2001; Yan and Caldwell 2001; Peng et al. 2015). Many adverse herb–drug interactions may be attributed to the inhibition of CYP450 enzymes by co-administrated drugs or herbs (Zhang et al. 2007; Nowack 2008; Jeong et al. 2013; Lee et al. 2013; Qi et al. 2013; Meng and Liu 2014). Therefore, the effects of bergenin on the activity of CYP enzymes should be investigated. To the best of our knowledge, few studies have investigated the effects of bergenin on CYP enzymes, particularly the inhibitory effects, which will increase the risk of therapeutic applications of bergenin and its medical preparations.

The purpose of this study was to investigate the effects of bergenin on eight major CYP isoforms in human liver microsomes (HLMs). *In vitro*, phenacetin (CYP1A2), testosterone (CYP3A4), coumarin (CYP2A6), chlorzoxazone (CYP2E1), dextromethorphan (CYP2D6), diclofenac (CYP2C9), S-mephenytoin (CYP2C19) and paclitaxel (CYP2C8) were used as probe substrates to determine the effects of bergenin on eight CYP enzymes. In addition, enzyme kinetic studies were conducted to determine the inhibition mode of bergenin on CYP enzymes.

## Materials and methods

### Chemicals

Bergenin (≥98%) and testosterone (≥98%) were obtained from the National Institute for the Control of Pharmaceutical and Biological Products (Beijing, China). d-Glucose-6-phosphate, glucose-6-phosphate dehydrogenase, corticosterone (≥98%), NADP^+^, phenacetin (≥98%), acetaminophen (≥98%), 4-hydroxymephenytoin (≥98%), 7-hydroxycoumarin (≥98%), 4′-hydroxydiclofenac (≥98%), sulphaphenazole (≥98%), quinidine (≥98%), tranylcypromine (≥98%), chlorzoxazone (≥98%), 6-hydroxychlorzoxazone (≥98%), paclitaxel (≥98%), 6β-hydroxytestosterone (≥98%), clomethiazole (≥98%) and furafylline (≥98%) were obtained from Sigma Chemical Co. (St. Louis, MO). Montelukast (≥98%) was obtained from Beijing Aleznova Pharmaceutical (Beijing, China). Coumarin (≥98%), diclofenac (≥98%), dextromethorphan (≥98%) and ketoconazole (≥98%) were purchased from ICN Biomedicals (Aurora, OH). Pooled HLMs were purchased from BD Biosciences Discovery Labware (Bedford, MD). All other reagents and solvents were of analytical reagent grade.

### Assay with human liver microsomes

As shown in Table 1, to investigate the inhibitory effects of bergenin on different CYP isoforms in HLM, the following probe reactions were used, according to a previously described method (Zhang et al. 2007; Qi et al. 2013): phenacetin *O*-deethylation for CYP1A2, testosterone 6β-hydroxylation for CYP3A4, coumarin 7-hydroxylation for CYP2A6, chlorzoxazone 6-hydroxylation for CYP2E1, dextromethorphan *O*-demethylation for CYP2D6, diclofenac 4′-hydroxylation for CYP2C9, *S*-mephenytoin 4-hydroxylation for CYP2C19 and paclitaxel 6α-hydroxylation for CYP2C8. All incubations were performed in triplicate, and the mean values were utilized. The typical incubation systems contained 100 mM potassium phosphate buffer (pH 7.4), NADPH generating system (1 mM NADP^+^, 10 mM glucose-6-phosphate, 1 U/mL of glucose-6-phosphate dehydrogenase and 4 mM MgCl_2_), the appropriate concentration of HLMs, a corresponding probe substrate and bergenin (or positive inhibitor for different probe reactions) in a final volume of 200 μL.

**Table 1.** Isoforms tested, marker reactions, incubation conditions and *K*_m_ used in the inhibition study.

CYPs	Marker reactions	Substrate concentration (μM)	Protein concentration (mg/mL)	Incubation time (min)	Estimated *K*_m_ (μM)
1A2	Phenacetin *O*-deethylation	40	0.2	30	48
3A4	Testosterone 6β-hydroxylation	50	0.5	10	53
2A6	Coumarin 7-hydroxylation	1.0	0.1	10	1.5
2E1	Chlorzoxazone 6-hydroxylation	120	0.4	30	126
2D6	Dextromethorphan *O*-demethylation	25	0.25	20	4.8
2C9	Diclofenac 4'-hydroxylation	10	0.3	10	13
2C19	S-Mephenytoin 4-hydroxylation	100	0.2	40	105
2C8	Paclitaxel 6α-hydroxylation	10	0.5	30	16

The concentration of bergenin was 100 μM, and the positive inhibitor concentrations were as follows: 10 μM furafylline for CYP1A2, 1 μM ketoconazole for CYP3A4, 10 μM tranylcypromine for CYP2A6, 50 μM clomethiazole for CYP2E1, 10 μM quinidine for CYP2D6, 10 μM sulphaphenazole for CYP2C9, 50 μM tranylcypromine for CYP2C19, 5 μM montelukast for CYP2C8. Probe substrates, positive inhibitors (except for dextromethorphan and quinidine which were dissolved in water) and bergenin were dissolved in methanol, with a final concentration of 1% (*v*/*v*), and 1% neat methanol was added to the incubations without inhibitor. The final microsomal protein concentration and incubation times for the different probe reactions are shown in Table 1. There was a 3 min pre-incubation period (at 37 °C) before the reaction was initiated by adding an NADPH-generating system. The reaction was terminated by adding a 100 μL acetonitrile (10% trichloroacetic acid for CYP2A6) internal standard mix, and the solution was placed on ice. The mixture was centrifuged at 12,000 rpm for 10 min, and an aliquot (50 μL) of the supernatant was transferred for HPLC analysis. The instrument used in this study were Agilent 1260 series instrument (Palo Alto, CA, USA) with DAD and FLD detector, and the quantitative assay for the corresponding metabolites was performed as previously reported (Lang et al. 2017; Zhang et al. 2017).

### Enzyme inhibition and kinetic studies of bergenin

A 100 μM bergenin was used to initially screen for its direct inhibitory effects towards different human CYP isoforms. For the CYP isoforms whose activities were strongly inhibited, secondary studies were performed to obtain the half inhibition concentration (IC_50_). *K_i_* values were obtained by incubating various concentrations of different probe substrates (20–100 μM testosterone, 25–200 μM chlorzoxazone or 2–20 μM diclofenac) in the presence of 0–50 μM bergenin.

### Time-dependent inhibition study of bergenin

To determine whether bergenin could inhibit the activity of CYP3A4, 2E1 and 2C9 in a time-dependent manner, bergenin (20 μM) was pre-incubated with HLMs (1 mg/mL) in the presence of an NADPH-generating system for 30 min at 37 °C. After incubation, an aliquot (20 μL) was transferred to another incubation tube (final volume 200 μL) containing an NADPH-generating system and probe substrates whose final concentrations were approximate to *K_m_*. Then, further incubations were performed to measure the residual activity. After being incubated for different time (30 min for CYP2E1 and CYP2C9, 10 min for CYP3A4), the reactions were terminated by adding a 100 μL acetonitrile internal standard mix and then placed on ice; the corresponding metabolites were determined by HPLC.

To determine the *K_I_* and *K*_inact_ values for the inactivation of CYP3A4, the incubations were conducted using higher probe substrate concentrations (approximately four-fold *K_m_* values) and various concentrations of bergenin (0–50 μM) after different pre-incubation times (0–30 min), with a two-step incubation scheme, as described above.

### Statistical analysis

The enzyme kinetic parameters for the probe reaction were estimated from the best fit line using least-squares linear regression of the inverse substrate concentration versus the inverse velocity (Lineweaver–Burk plots), and the mean values were used to calculate *V*_max_ and *K_m_*. Inhibition data from the experiments that were conducted using multiple compound concentrations were represented by Dixon plots, and inhibition constant (*K_i_*) values were calculated using non-linear regression according to the following equation:

$$v\;=\;\left(V_{\text{max}}S\right)/\left(K_{m}\left(\text{1 }+\;I/K_{i}\right)\;+\;S\right),$$

where *I* is the concentration of the compound, *K_i_* is the inhibition constant, *S* is the concentration of the substrate and *K_m_* is the substrate concentration at half the maximum velocity (*V*_max_) of the reaction. The mechanism of the inhibition was inspected using the Lineweaver–Burk plots and the enzyme inhibition models. The data comparison was performed using the Student’s *t*-test and performed using IBM SPSS statistics 20 (SPSS Inc., Chicago, IL).

## Results

**Figure 1.**
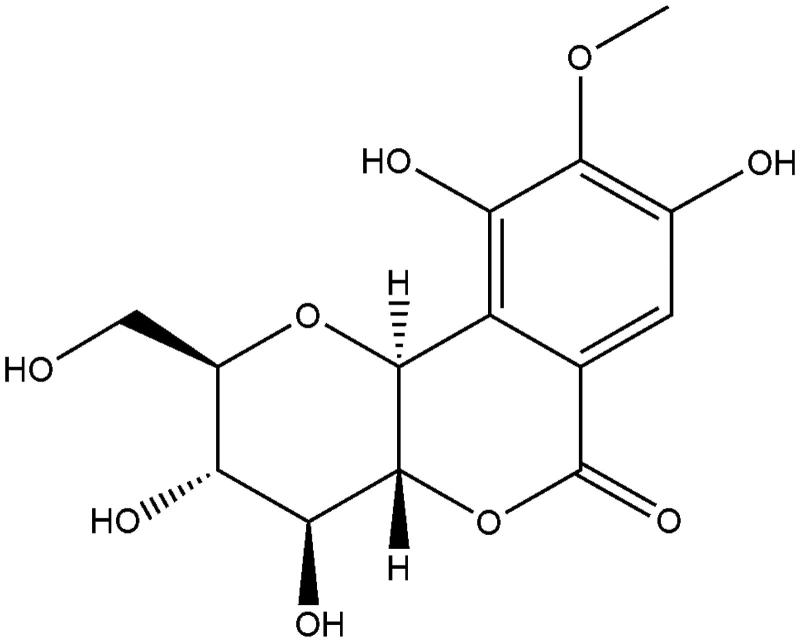
The chemical structure of bergenin.

**Figure 2.**
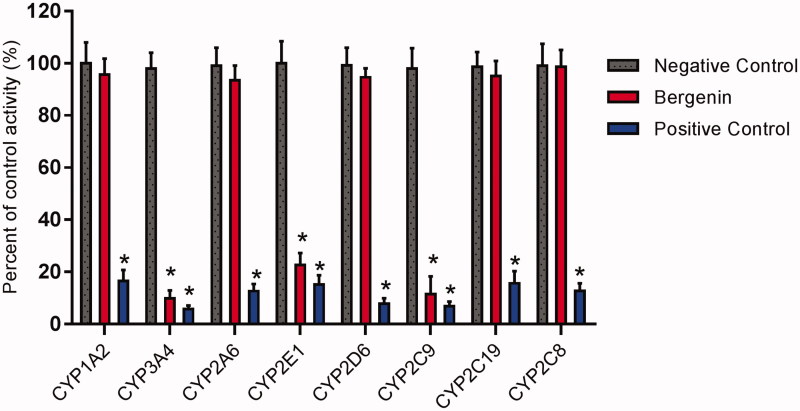
Effects of bergenin (100 μM) on the activity of CYP450 enzymes in pooled HLMs. All data represent mean ± S.D. of the triplicate incubations. *p < 0.05, significantly different from the negative control. Negative control: incubation systems without bergenin; bergenin: incubation systems with bergenin; positive control: incubation systems with their corresponding positive inhibitors.

To investigate whether the bergenin affects the catalytic activity of CYP enzymes, the probe reaction assays were conducted with varying concentrations of bergenin (Figure 1). Specific inhibitors of CYP1A2, 3A4, 2A6, 2E1, 2D6, 2C9, 2C19 and 2C8 were used as positive controls. As shown in Figure 2, bergenin did not inhibit the activities of CYP1A2, 2A6, 2D6, 2C19 and 2C8 at a concentration of 100 μM. In contrast, the activities of CYP1A2, 3A4 and 2E1 were inhibited to 25.8, 11.0 and 12.2% of their control activities, respectively.

The enzyme-inhibition study showed that inhibition of CYP3A4, 2E1 and 2C9 by bergenin was concentration-dependent, with IC_50_ values of 14.39, 22.83 and 15.11 μM, respectively.

**Figure 3.**
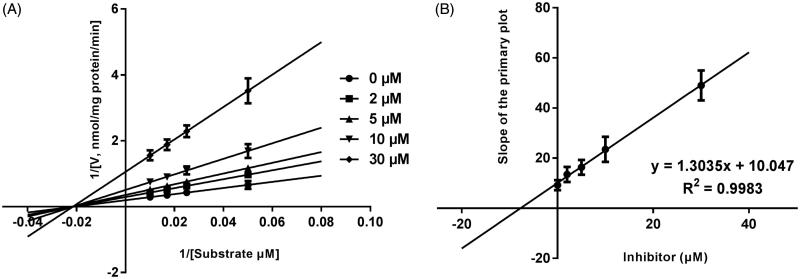
Lineweaver–Burk plots (A) and the secondary plot for *K_i_* (B) of effects of bergenin on CYP3A4 catalyzed reactions (testosterone 6β-hydroxylation) in pooled HLM. Data were obtained from 30 min incubation with testosterone (20–100 μM) in the absence or presence of bergenin (0–30 μM). All data represent mean ± S.D. of the triplicate incubations.

**Figure 4.**
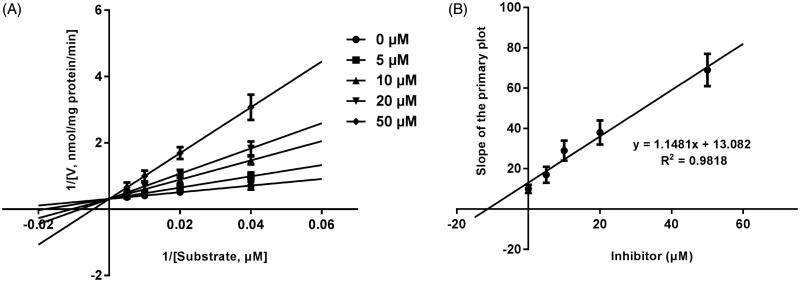
Lineweaver–Burk plots (A) and the secondary plot for *K_i_* (B) of effects of bergenin on CYP2E1 catalyzed reactions (chlorzoxazone 6-hydroxylation) in pooled HLM. Data were obtained from 30 min incubation with diclofenac (25–250 μM) in the absence or presence of bergenin (0–50 μM). All data represent mean ± S.D. of the triplicate incubations.

**Figure 5.**
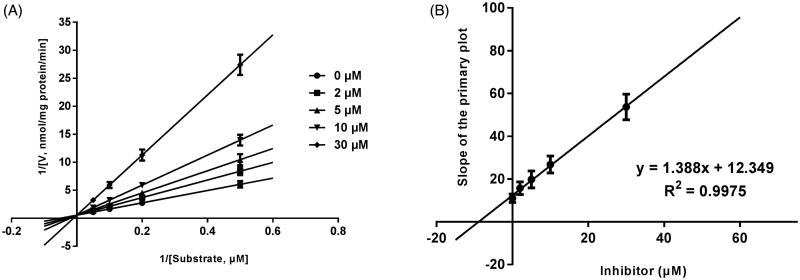
Lineweaver–Burk plots (A) and the secondary plot for *K_i_* (B) of effects of bergenin on CYP2C9 catalyzed reactions (diclofenac 4′-hydroxylation) in pooled HLM. Data were obtained from 30 min incubation with phenacetin (2–20 μM) in the absence or presence of bergenin (0–30 μM). All data represent mean ± S.D. of the triplicate incubations.

Lineweaver–Burk plots of inhibitory kinetic data suggested that the inhibition of the inhibition of CYP3A4 by bergenin was best fit in a non-competitive manner (Figure 3(A)), whereas CYP2E1 (Figure 4(A)) and 2C9 (Figure 5(A)) by bergenin was the best fit in a competitive manner. The *K_i_* values of bergenin on CYP3A4 (Figure 3(B)), 2E1 (Figure 4(B)) and 2C9 (Figure 5(B)) were obtained from the secondary Lineweaver–Burk plot for *Ki*, with values of 7.71, 11.39 and 8.89 μM, respectively.

**Figure 6.**
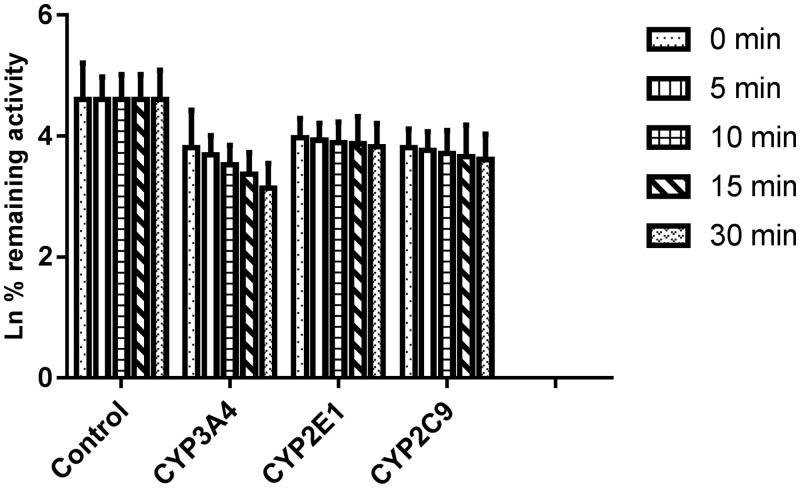
Time-dependent inhibition investigations of CYP3A4, 2E1 and 2C9 catalyzed reactions by bergenin (20 μM). All data represent mean ± S.D. of the triplicate incubations.

**Figure 7.**
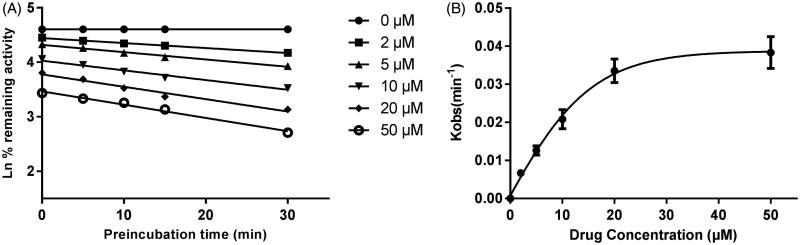
Time and concentration-inactivation of microsomal CYP3A4 activity by bergenin in the presence of NADPH. The initial rate constant of inactivation of CYP3A4 by each concentration (*K*_obs_) was determined through linear regression analysis of the natural logarithm of the percentage of remaining activity versus pre-incubation time (A). The *K_I_* and *K*_inact_ values were determined through non-linear analysis of the *K*_obs_ versus the bergenin concentration (B).

As shown in Figure 6, after pre-incubation of bergenin with HLM for 30 min, the activity of CYP3A4 decreased with the incubation time, however, the activity of CYP2E1 and 2C9 was not affected. To characterize the time-dependent inhibition of CYP3A4 by bergenin, inactivation parameters of *K_I_* and *K*_inact_ values were calculated using non-linear regression analysis in HLM. As calculated from the inactivation plot of Figure 7, the *K*_inact_/*K_I_* value for CYP3A4 was 0.025/3.50 μM^−1 ^min^−1^. The *K*_inact_ values imply that approximately 2.5% of CYP3A4 is inactivated each minute when a saturating concentration of bergenin is incubated with HLM.

## Discussion

In clinical practice, many patients undergo multiple-drug therapy. Multiple-drug therapy possesses several advantages, such as simultaneously treatment of diseases, or multi-drug therapy for the treatment of complex chronical disorders, resulting in a better treatment outcome compared with monotherapy. However, many herb–drug interactions resulting from the concurrent use of herbal drugs with prescription and over-the-counter drugs may cause adverse reactions such as toxicity and treatment failure (Zhou et al. 2004). The most common causes of herb–drug interactions are a modification of the enzyme activity of cytochrome P450 enzymes, specifically through inhibitory effects. Inhibition of CYP enzymes *in vivo* may result in unexpected elevations in the plasma concentrations of concomitant drugs, leading to adverse effects (Hu et al. 2015; Liu et al. 2015). Therefore, regulatory authorities require preclinical (*in vitro*) and clinical (*in vivo*) interaction studies in the drug development.

As bergenin possesses numerous pharmacological activities and has the potential of becoming a lead compound of anticancer drugs, it is essential to investigate the inhibitory effects of bergenin on the major CYP enzymes. To the best of our knowledge, this study is the first to investigate the effects of bergenin on the metabolism of probe substrates of several CYP isoforms, including CYP1A2, 3A4, 2A6, 2E1, 2D6, 2C9, 2C19 and 2C8.

The CYP3A subfamily is one of the dominant CYP enzymes in the liver and extra-hepatic tissues, such as the intestines, and it plays an important role in the oxidation of xenobiotics and contributes to the biotransformation of approximately 60% of currently used therapeutic drugs (Pandit et al. 2011). Human CYP3A4 is one of the most abundant drug-metabolizing CYP isoforms in human liver microsomes, accounting for approximately 40% of the total CYP enzymes (Zhou 2008). In fact, characterization of the CYP3A4 isoform responsible for the metabolism of drugs and herbal constituents is important for identifying potential drug–drug or herb–drug interactions in humans. The present study showed that bergenin had inhibitory effects *in vitro* on CYP3A4 isoform, with *K_i_* and IC_50_ values of 7.71 μM and 14.39 μM, respectively. The results suggested that bergenin was also a weak CYP3A4 inhibitor, and the potential of herb–drug interaction with CYP3A4 would also be low. However, the results also indicated that bergenin is a time-dependent inhibitor for CYP3A4 with *K*_inact_/*K_I_* value of 0.025/3.50 μM^−1 ^min^−1^, which revealed that bergenin would inhibit the activity of CYP3A4 with an increase of pre-incubation time of concentration. Therefore, to avoid adverse drug interactions, bergenin should not be used with other drugs metabolized by CYP3A4.

CYP2E1 also play an important role in the metabolism of many drugs (Sun et al. 2014; Bedada and Neerati 2018). Our study showed that bergenin competitively inhibited human liver microsomal CYP2E1 activity. Therefore, bergenin should also be used carefully with drugs metabolized by CYP2E1 to avoid possible drug interactions even though bergenin was an inhibitor for these two CYP isoforms.

Our study showed that bergenin competitively inhibited human liver microsomal CYP2C9 activity. Therefore, bergenin should also be used carefully with drugs metabolized by CYP2C9 to avoid possible drug interactions even though bergenin was an inhibitor for these two CYP isoforms.

As we know, *in vitro* data are essential for understanding a potential enzyme inhibition and DDI *in vivo*. However, an observed *in vitro* inhibition of a CYP enzyme does not mean that the drug will cause clinically relevant interactions. Many other factors might influence drug interactions mediated by CYP inhibition, including the contribution of the hepatic clearance to the total clearance of the affected drug, the fraction of the hepatic clearance which is subject to metabolic inhibition, and the ratio of the inhibition constant (*Ki*) over the *in vivo* concentration of the inhibitor (Ito et al. 1998; Ericsson et al. 2014). Therefore, further *in vivo* system studies are needed to identify the interactions of bergenin with CYP isoform in humans.

The results of this study indicate that bergenin may influence the *in vitro* metabolism of drugs that are substrates of CYP3A4, 2E1 and 2C9. Pan et al. (2016) have reported that the plasma *C*_max_ values of bergenin in rats treated with an oral dose (100 mg/kg) were less than 300 ng/mL (0.9 μM). Therefore, the rat plasma concentrations of bergenin were much lower than the *K_i_* and IC_50_ values of bergenin determined in this study, and severe drug–drug interaction might not occur if bergenin were co-administered with the substrates of the CYP3A4, 2E1 and 2C9. However, due to the pharmacokinetic differences of human and rats, further *in vivo* system studies are needed to identify the interactions of bergenin with CYP isoform in humans.

In conclusion, the effects of bergenin on the activity of CYP enzymes were systematically investigated. The results showed that bergenin could inhibit the activity of CYP3A4, 2E1 and 2C9, while the activities of other CYP enzymes were not affected. Therefore, to avoid adverse drug interactions, it is recommended that bergenin should not be used with other drugs metabolized by CYP3A4, 2E1 and 2C9.
